# Account of Diseases in an Eastern District of London, from Nov. 20, to Dec. 20, 1802

**Published:** 1803-01-01

**Authors:** 


### *Account of Diseases in an Eastern District of London, from Nov. 20, to Dec. 20, 1802.*

| ACUTE DISEASES. | |
|---|---|
| Typhus Mitior | 3 |
| Peripneumonia Notha | 4 |
| Scarlatina Anginosa | 8 |
| Cynanche Tonsillaris | 3 |
| Rheumatismus Acutus | 4 |

| CHRONIC DISEASES. | |
|---|---|
| Tussis | 20 |
| Tussis cum Dyspnœa | 16 |
| Dyspnœa | 10 |
| Catarrhus | 7 |
| Pleurodyne | 6 |
| Phthisis Pulmonalis | 5 |
| Hydrothorax | 2 |
| Palpitatio Cordis | 1 |
| Ascites | 2 |
| Anasarca | 3 |
| Vertigo | 4 |
| Paralysis | 2 |
| Amenorrhœa | 7 |
| Chlorosis | 5 |
| Nephralgia | 2 |
| Menorrhagia | 3 |
| Fluor Albus | 5 |
| Gastrodynia | 7 |
| Rheumatismus Chronicus | 12 |

| PUERPERAL DISEASES. | |
|---|---|
| Menorrhagia Lochialis | 3 |
| Rhagas Papillæ | 2 |
| Enuresis | 1 |

| INFANTILE DISEASES. | |
|---|---|
| Ophthalmia | 3 |
| Aphthæ | 4 |
| Dentition | 1 |

The Scarlatina Anginosa still continues to prevail, particularly amongst the younger branches of any family where it occurs. It has indeed been so general, that very few of those who have continued in the house have entirely escaped it. This remark applies, more particularly, to the families of the poor, as, in course, every disease which has a tendency to propagate itself, must spread more rapidly where the subjects of it are confined within a narrow space, and where fresh air is supplied in a scanty portion. This disease has been very different in the degree of its violence. In some cases it has been so slight as hardly to require any medical assistance, but in others so severe as to excite considerable alarm.

Some of the patients have complained of a great soreness of the mouth, but this has sometimes attended a mild form of the disease ; and though very troublesome, has been frequently accompanied by a mitigation of other symptoms. In one instance, which occurred to the observation of the Reporter, the appearance of some aphthous ulceration about the tongue and fauces was a prelude to the favourable termination of the disease, which had before assumed an unpleasant appearance, and excited considerable anxiety.

